# Immunomodulatory Properties of PI3K/AKT/mTOR and MAPK/MEK/ERK Inhibition Augment Response to Immune Checkpoint Blockade in Melanoma and Triple-Negative Breast Cancer

**DOI:** 10.3390/ijms23137353

**Published:** 2022-07-01

**Authors:** Zhizhu Zhang, Ann Richmond, Chi Yan

**Affiliations:** 1 Department of Veterans Affairs, Tennessee Valley Healthcare System, Nashville, TN 37212, USA; zhizhu.zhang@vanderbilt.edu (Z.Z.); ann.richmond@vanderbilt.edu (A.R.); 2 Department of Pharmacology, Vanderbilt University School of Medicine, Nashville, TN 37240, USA

**Keywords:** melanoma, triple-negative breast cancer, PI3K/AKT/mTOR, MAPK/MEK/ERK, immune checkpoint blockade

## Abstract

Hyperactivation of PI3K/AKT/mTOR and MAPK/MEK/ERK signaling pathways is commonly observed in many cancers, including triple-negative breast cancer (TNBC) and melanoma. Moreover, the compensatory upregulation of the MAPK/MEK/ERK pathway has been associated with therapeutic resistance to targeted inhibition of the PI3K/AKT/mTOR pathway, and vice versa. The immune-modulatory effects of both PI3K and MAPK inhibition suggest that inhibition of these pathways might enhance response to immune checkpoint inhibitors (ICIs). ICIs have become the standard-of-care for metastatic melanoma and are recently an option for TNBC when combined with chemotherapy, but alternative options are needed when resistance develops. In this review, we present the current mechanistic understandings, along with preclinical and clinical evidence, that outline the efficacy and safety profile of combinatorial or sequential treatments with PI3K inhibitors, MAPK inhibitors, and ICIs for treatment of malignant melanoma and metastatic TNBC. This approach may present a potential strategy to overcome resistance in patients who are a candidate for ICI therapy with tumors harboring either or both of these pathway-associated mutations.

## 1. Introduction

**Figure 1:**
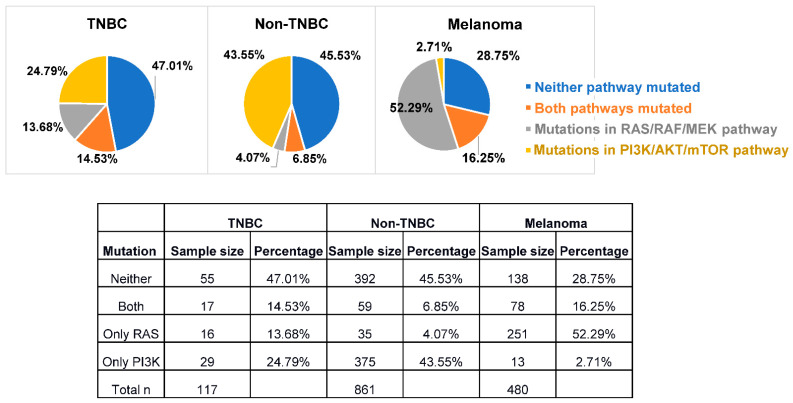
**Percentage of TNBC, non-TNBC, and melanoma patients with mutations in the PI3K and MAPK pathways.** Mutations in each pathway were identified as somatic genetic variant in at least one gene from the PI3K pathway (*PTEN, PIK3CA, AKT1, ERBB2, PIK3R1*) or RAS pathway (*HRAS, KRAS, NRAS, BRAF, NF1,* and *SOS1*). TNBC, triple-negative breast cancer.

Aberrant activation of the phosphoinositide 3-kinase (PI3K) and mitogen-activated protein kinase (MAPK) pathways play an important role in the development, progression, and drug resistance mechanisms of multiple cancers. Melanoma and breast cancer frequently harbor genetic alterations associated with the PI3K and MAPK pathways (Figure 1), which often lead to the upregulation of pathway activation. While dual inhibition of BRAF and MEK as a standard treatment for advanced BRAF-mutant melanoma showed a high response rate, the response duration for many patients is relatively short [1]. In addition, PI3K inhibitors as monotherapy showed limited efficacy in clinical trials [2]. The compensatory mechanisms and feedback loops between the PI3K/AKT/mTOR and MAPK/MEK/ERK pathways may underlie intrinsic or acquired resistance to each of these pathway’s targeted inhibition [3].

The recent approval of immune checkpoint inhibitors (ICIs) offered new options for melanoma and triple-negative breast cancer (TNBC) patients, with better response rates in melanoma than in breast cancer [4,5]. Many hypothesize that this discrepancy is partly due to an immunosuppressive tumor microenvironment. In addition to their direct effect on tumors, PI3K/AKT/mTOR and MAPK/MEK/ERK inhibitors have been shown to modulate the tumor microenvironment, increase immunogenicity, and thus potentially facilitate increased sensitivity to immunotherapy. Understanding the interplay between PI3K and MAPK cascades and their roles in tumor immunity is essential for extending the clinical benefit of existing treatments to a larger patient population. This review will discuss the feasibility of combining MAPK/MEK/ERK and PI3K/AKT/mTOR targeted therapy to overcome resistance to immunotherapy. Specifically, the combination or sequential treatments could benefit TNBC and melanoma patients with mutations in both pathways, rendering them prone to monotherapy resistance.

**Figure 2:**
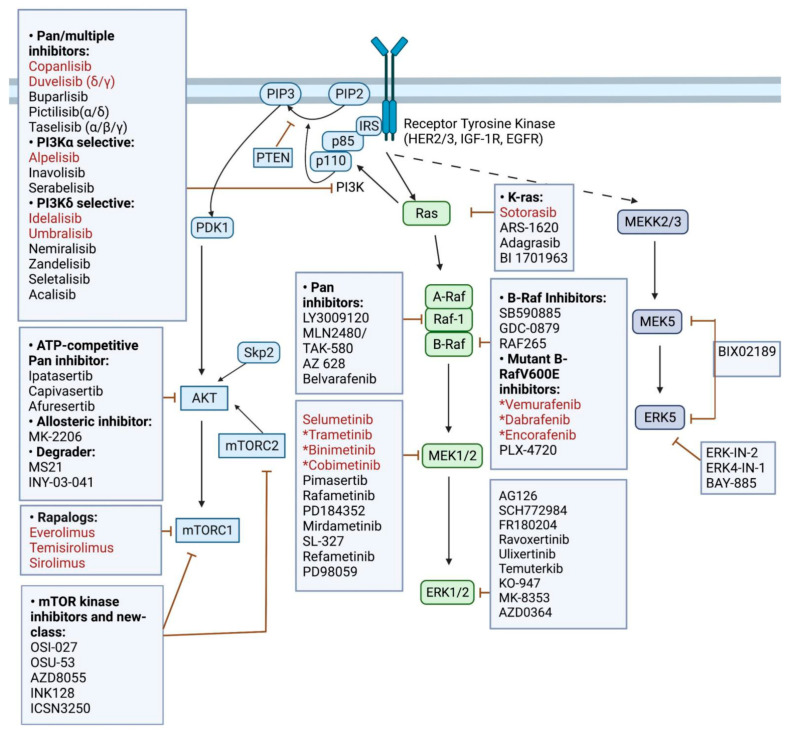
**The overview of PI3K/AKT/mTOR, RAS-RAF-MEK-ERK, and MEK5/ERK5 pathway, with their respective small molecule inhibitors**. The immune-modulatory effect of both PI3K and MAPK inhibition provides a promising option to overcome therapeutic resistance with ICIs. Dual-targeted inhibition of MAPK and PI3K pathway effectors in combination or with sequential treatment with ICIs may present a potential strategy to overcome resistance in patients with tumors harboring both pathway-associated mutations. FDA-approved therapeutic agents are denoted in red. Inhibitors with an asterisk (*) are approved in melanoma. Created with BioRender.com.

A comprehensive literature search was performed to identify published scientific research and clinical trials employing targeted inhibitors of MAPK/MEK/ERK and/or PI3K/AKT/mTOR combined with immunotherapies as of 2 April, 2022. PubMed, ClinicalTrials.gov, ASCO, AACR, and ESCO meeting abstracts were searched. Keywords included melanoma, TNBC, immunotherapies, PD-1, PD-L1, CTLA-4, RAS, BRAF, MEK, ERK, PI3K, AKT, mTOR, the targeted inhibitors listed in Figure 2, and ICIs (ipilimumab, nivolumab, pembrolizumab, atezolizumab, spartalizumab, durvalumab, and their alternative names). In addition, some references of included trials and relevant reviews were traced for manual review.

### 1.1. Overview of the PI3K and MAPK Signaling Pathways and Their Inhibitors

The PI3K/AKT/mTOR pathway is involved in cell growth, apoptosis regulation, and glucose metabolism, among many other pro-tumor processes [6]. Signaling from RAS or directly from the receptor tyrosine kinases (RTKs) results in active catalysis of PI3K, leading to the activation of AKT. AKT can then promote multiple downstream signaling cascades, including the mammalian target of rapamycin (mTOR) (Figure 2). As mTOR binds to other protein partners in mammalian cells, it forms two distinct complexes, mTORC1 (mTOR complex 1) and mTORC2 (mTOR complex 2) [7]. mTORC1 activation induces mRNA translation of the biosynthesis machinery to promote cell growth, while mTORC2 can phosphorylate AKT to enhance its catalytic activity in a positive feedback loop [7,8]. Two common types of genetic aberrations in the PI3K pathway are mutations in the PIK3CA gene, which encodes the p110α catalytic subunit of PI3K and inactivating mutations or genetic loss of the negative regulator of the PI3K/AKT/mTOR pathway, phosphate and tensin homolog (PTEN) [9]. PTEN deletion or inactivating mutations are detected in 18% of TNBC patients, and PIK3CA mutations are observed in 16% of patients [n = 116, TCGA Firehose Legacy (cBioportal.org)]. In melanoma, PTEN deletion or inactivating mutations are found in 12% of patients, and 3% of patients carried PIK3CA mutations [n = 471, TCGA-melanoma Firehose Legacy (cBioportal.org)]. However, activation of numerous tyrosine kinase receptors expressed on both melanoma and TNBC tumors leads to activation of both the PI3K/AKT/mTOR and MAPK pathways [10,11].

Multiple targeted inhibitors for PI3K, AKT, or mTOR have been developed (Figure 2). For example, PI3K inhibitors can be broadly acting (e.g., copanlisib and duvelisib) or isoform-specific (e.g., alpelisib). ATP-competitive AKT inhibitors, such as ipatasertib and capivasertib, and the allosteric AKT1/2 inhibitor MK-2206 [12], have been tested in various phase Ib/II clinical trials. mTOR rapalogs, such as everolimus and temsirolimus, preferentially inhibit mTORC1, while mTOR kinase inhibitors target both mTORC1 and mTORC2.

A complementary signaling pathway to PI3K/AKT/mTOR is the MAPK/MEK/ERK pathway (Figure 2). MAPK signaling also plays a crucial role in multiple cellular processes, including cell proliferation, survival, and gene expression. Upon dimerization of RTKs, activation of RAS occurs via the GTP exchange mechanism [13]. This RAS activation triggers the dimerization of CRAF and BRAF, leading to phosphorylation of downstream kinases MEK1/2 [14]. Phospho-MEK1/2 then activates ERK1/2, thereby inducing a cascade of substrates, such as cyclin D and CDK4/6, to promote the G1/S phase transition [15]. Upregulation of this pathway is found in multiple cancers, promoting cell cycle progression, resistance to intrinsic apoptosis, and epithelial-mesenchymal transition (EMT) [16]. Activating mutations of NRAS, KRAS, HRAS, and BRAF enable constitutive activation of downstream signaling [16]. Approximately 20% of TNBC patients have a mutation in HRAS, KRAS, BRAF, or NF1 genes [(n = 116, TCGA Firehose Legacy, (cBioportal.org)]. In contrast, 67% of melanoma patients have a mutation in KRAS, NRAS, BRAF, or NF1 genes (n = 471). Additionally, 14.53% of TNBC patients and 16.25% of melanoma patients exhibited mutations in both MAPK and PI3K pathways (Figure 1).

Potent and selective small-molecule inhibitors of the RAS/RAF/MEK pathway have also been extensively explored in anti-cancer treatments (Figure 2). First-generation Raf kinase inhibitors, such as vemurafenib, dabrafenib, and encorafenib, are selective to mutant BRAF^V600E^. However, recovery of ERK activation due to Raf dimerization could lead to a rapid development of resistance upon inhibition of BRAF [17]. Combining BRAF inhibitors with MEK1/2 inhibitors, such as trametinib, binimetinib and cobimetinib, enhances the durability of the efficacy. Additionally, novel pan-Raf inhibitors, such as LY3009120 and TAK-580, inhibit both monomeric and dimeric forms of Raf to overcome resistance. Additionally, inhibitors of the downstream effector ERK, such as ulixertinib, are under clinical investigation.

### 1.2. Compensatory MAPK Signaling in PI3K/AKT/mTOR Inhibition Resistance and Dual Inhibition Therapies

Despite the development of multiple PI3K-targeted inhibitors (Figure 2), use as monotherapy in TNBC and melanoma has shown limited clinical benefits due to intrinsic and acquired resistance. For example, inhibition of PI3K can result in compensatory insulin release, which hyperphosphorylates the insulin growth factor receptor (IGF1R). Subsequently, recruited insulin receptor substrate (IRS) adaptor molecules reactivate the PI3K signaling axis in tumors and rescue AKT and S6 phosphorylation [18]. This insulin-mediated feedback loop can circumvent the inhibitory effects of PI3Kα inhibitors, mTORC1 inhibitors, and AKT inhibitors [19,20,21]. In addition, loss of PTEN expression resulting from DNA copy number loss and genetic alterations has been associated with acquired resistance to PI3Kα inhibition. Still, resistance can be subverted through concurrent inhibition of α and β subtypes of the p110 PI3K subunit [22]. Though mTOR kinase and AKT inhibitors temporarily pause PI3K signaling, resistance can develop quickly via the abrogation of feedback inhibition on RTKs, restoring PI3K activity [19,23,24]. AKT can also be reactivated independently of PI3K through the S-phase kinase-associated protein 2 (Skp2) [23]. Breast cancer cell lines with diminished PI3K activity via PIK3CA depletion or treatment with pan-PI3K inhibitor BKM120 exhibited an increase in ubiquitin E3 ligase Skp2, resulting in non-canonical AKT rebound activation [23].

The inhibitory effects of PI3K/AKT/mTOR inhibition are often negated via upregulation of parallel signaling pathways, including the compensatory activation of MAPK signaling. The PI3K and MAPK pathways are both downstream of RTKs/RAS and activated by secondary messengers like PIP2. Thus, single inhibition of either pathway could bring compensatory propagation of the other pathway, countering the desired suppression of tumor growth, proliferation, and survival. For instance, inhibition of mTORC1 enhances the phosphorylation of ERK on the activation loop residues in breast and melanoma patients under treatment [25]. Furthermore, phospho-proteome and kinome analysis of TNBC patient-derived xenografts demonstrated significantly upregulated MAPK signaling following treatment with and resistance to pan-PI3K inhibitor buparlisib [26]. In addition, the combination of buparlisib and a MEK inhibitor trametinib has a synergistic anti-tumor effect, confirming that crosstalk between PI3K/AKT/mTOR and MAPK/MEK/ERK signaling may contribute to PI3K inhibitor resistance [26].

Excessive activation of the PI3K pathway has also been identified as a resistance mechanism to MAPK inhibition. PTEN loss and the subsequent increase in AKT signaling limits BRAF inhibitor-induced apoptotic responses in melanoma. However, this was overcome with the co-administration of a PI3K inhibitor [27]. Vemurafenib (BRAF inhibitor)-resistant melanoma cell lines showed increased phospho-AKT levels, while the addition of either AKT or mTORC1 inhibitors led to the reversal of resistance [28].

Several preclinical studies confirmed that dual pharmacological inhibition of PI3K and MAPK pathways (via both continuous and intermittent dosing) improved therapeutic activity in basal-like breast cancer and melanoma models, as well as cell lines with mutations in both pathways [29,30,31]. In addition, combined treatment reduced tumor growth via the induction of multiple proapoptotic biomarkers [29].

Nevertheless, toxicity has limited the success of the dual combination in multiple recent clinical trials. Dose interruptions, reduction, and discontinuation of treatment frequently occur with hematologic, gastrointestinal, and dermatologic adverse events (AEs). A phase Ib study (NCT01390818) tested MEK inhibitor pimasertib and PI3K/mTOR inhibitor voxtalisib in patients with advanced solid tumors, including TNBC and BRAF^V600^-mutant melanoma patients who progressed on BRAF inhibitors [32]. Unfortunately, low tolerability and limited clinical efficacy prevented it from progressing into further testing. Similar dose-limiting toxicities were found with the pan-PI3K inhibitor BKM120 and the BRAF inhibitor vemurafenib in BRAF^V600^-mutant advanced melanoma patients (NCT01512251) [33]. The use of pan-AKT inhibitor uprosertib with MEK1/2 inhibitor trametinib was also not well-tolerated, with minimal clinical advantage in continuous or intermittent dosing for patients with TNBC or BRAF^WT^ melanoma (NCT01138085) [34]. The maximum tolerated dose (MTD) for uprosertib was, at most, 67% of the target dose in monotreatment with combined continual dosing, and intermittent dosing also could not bring higher exposure levels within tolerable ranges [34]. The only study with promising results investigated the combination of BRAF inhibitor vemurafenib and mTOR inhibitor everolimus in 18 patients with BRAF-mutated advanced solid tumors (NCT01596140) [35]. The toxicity profile of this study was tolerable, with 22% partial response (PR) and 50% stable disease (SD), though the patient population was small and highly heterogeneous [35]. Notably, a patient with metastatic PTEN(P95S)-mutated melanoma who progressed on previous treatment of vemurafenib and PI3K inhibitor (PX866) achieved PR [35]. The safety and efficacy of this combination study highlighted the complexity and importance of inhibitor selection within the two pathways and the optimal patient population.

### 1.3. The rationale for Combined Immune Checkpoint Blockade and Targeted Therapy 

Aside from the direct anti-proliferative effect on tumor cells, increasing evidence supports the immune-modulatory effect of PI3K/AKT/mTOR and MAPK/MEK/ERK inhibitors, making their adjuvant potential with immunotherapy an intriguing therapeutic option. Our group recently conducted a review of the immunosuppressive role of PI3K signaling, including the PTEN-loss-associated increase in PD-L1 expression, the recruitment of myeloid-derived suppressor cells (MDSC) and regulatory T cells (Treg), and the rationale for combining PI3K/AKT/mTOR inhibition with immunotherapy to reverse therapeutic resistance [36]. While treatment with PI3Kα specific or pan-PI3K inhibitor in murine TNBC models did not improve anti-tumor response over ICI alone, the PI3K/mTOR dual inhibitor gedatolisib synergized with ICIs, leading to greater growth inhibition with increased activation of T-cell, natural killer (NK)-cell, and dendritic cell (DC) responses [37]. Results from the combination of durvalumab (anti-PD-L1) with paclitaxel and capivasertib (AKT inhibitor) as first-line treatment for patients with metastatic PD-L1^+^ TNBC in the phase 1b/2 BEGONIA trial were recently reported [38]. The objective response rate (ORR) is numerically similar [16/30 (53.3%) vs. 13/23 (56.5%)], though the small sample size did not allow effective comparison across treatment groups. The grade 3–4 treatment-related AEs were relatively higher for the triplet treatment compared to the durvalumab/paclitaxel doublet, but the discontinuation rate for AEs was lower [38]. Another phase II study (Mario-3, NCT03961698) evaluated the triplet combination of eganelisib, a PI3K-γ inhibitor, with atezolizumab and nab-paclitaxel as first-line therapy for locally advanced or metastatic TNBC patients, reporting an ORR of 55.3% irrespective of PD-L1 status and tolerable toxicity [39]. A phase Ib study of anti-PD1 antibody spartalizumab and everolimus in TNBC (NCT02890069) recently closed but has not yet published the results. Other trials are still ongoing (Table 1). 

Similar to the PI3K pathway, constitutive activation of MAPK signaling has been associated with features of immune evasion, including increased infiltration of Tregs, accumulation of MDSCs, downregulation of tumor antigen presentation, reduced release of effector cytokines such as IFNγ, IL-2, and TNFα, and enhanced expression of immunosuppressive IL-10 and CCL2 [40]. In preclinical studies, inhibition of the MAPK pathway has been shown to reverse these immunosuppressive processes. Restoration of DC function, increased CD8^+^ T-cell and NK-cell infiltration, T-cell cytotoxicity (perforin, granzyme B), and melanoma antigen presentation, with a decrease in IL-6, IL-8, and CCL2 were observed in mouse melanoma models and patient biopsies treated with a BRAF inhibitor with or without concurrent MEK inhibition [41,42,43]. These studies provide a rationale for combining MAPK/MEK/ERK targeted therapy with immunotherapy to prevent immune escape and maintain therapeutic responses in cancer patients. Indeed, multiple preclinical studies in murine melanoma models examined the combination of BRAF/MEK inhibition with anti-PD-1/PD-L1 and showed promising results. For instance, in anti-PD-1 resistant BRAF-mutant melanoma models, concurrent treatment with dabrafenib and trametinib led to significantly enhanced tumor growth inhibition, increased CD8^+^ cytotoxic and CD4^+^ helper T cell infiltration, and an upregulation of MHC class I and II molecules [44,45].

Interestingly, clinical trials using the concomitant treatment of RAF/MEK inhibitors and ICI showed mixed results. Phase I studies of concurrent administration of ipilimumab, an anti-CTLA-4 antibody, with RAF/MEK inhibitors showed severe hepatotoxicity or gastrointestinal toxicity that led to the termination of the trials [46,47]. In Phase III clinical trial IMspire150 (NCT02908672), previously untreated, advanced, unresectable BRAF^V600E^ mutant melanoma patients were first treated with targeted therapy of vemurafenib plus cobimetinib for 21 days, followed by vemurafenib monotherapy for seven days, then the triple combination with PD-L1 inhibitor atezolizumab from cycle two onwards. The control group received an intravenous placebo and vemurafenib + cobimetinib combination [48]. Grade 3-4 treatment-related adverse events occurred in 79% of patients in triplet therapy vs. 73% in doublet therapy. Based on the prolonged median progression-free survival (PFS) (15.1 mo vs. 10.6 mo), the triple combination gained FDA approval [48]. However, other clinical trials with similar combination strategies reported numerically increased but statistically insignificant survival benefits. A phase I/II combination trial with dabrafenib, trametinib, and pembrolizumab in BRAF^V600E^-mutant metastatic melanoma patients (KEYNOTE-22, NCT02130466) did not reach its primary endpoint, despite a subset of patients who benefited from a long-duration response [49]. 58% (vs. 25% in doublet therapy) of patients had grade 3-5 treatment-related AEs [49]. The Phase III clinical trial COMBI-i (NCT02967692) examined at anti-PD1 antibody, spartalizumab, combined with full doses of dabrafenib and trametinib [50]. The study did not show a statistically significant difference in PFS (16.2 mo vs. 12.0 mo) or objective response rate (69% vs. 64%), while grade ≥three treatment-related AEs are more frequent in patients in the triplet treatment (55% vs. 33%) [50]. The mixed results of concomitant treatment of RAF/MEK inhibitors and ICI may be due to the choice of therapeutic agents (anti-PD-L1 in IMspire150 vs. anti-PD1 in COMBI-i), the design of initial targeted treatment run-in in IMspire150, patient population, and/or statistical parameters. These studies suggest only a moderate benefit of RAF/MEK inhibitors and ICI concomitant treatment, with an increased toxicity profile. Currently, other clinical trials are ongoing to explore the potential improvement in clinical outcomes (Table 1). **Table 2** summarizes the reported results from clinical trials combining immunotherapy and targeted therapies in BRAF/MEK or PI3K inhibition in melanoma or TNBC patients.

### 1.4. Treatment Sequence of Combined Immunotherapy and Targeted BRAF/MEK Inhibition

While the approval of both immunotherapy and BRAF/MEK inhibition as first-line treatments expanded options for metastatic melanoma, the toxicity profile of concomitant treatment with a modest response rate was discouraging. Alternatively, a sequential treatment strategy could potentially decrease adverse events and improve the durability of the therapy. Specifically, it is important to elucidate whether BRAF/MEK targeted therapy (TT) should precede or follow immunotherapy (IT).

Frederick et al. reported that tumor biopsies following BRAF ± MEK inhibitor combination therapy exhibited an increase in T-cell exhaustion markers (TIM3 and PD1) and downregulation of both melanoma antigen presentation and CD8^+^ T cell infiltration upon progression on BRAF inhibitors [41]. These results suggest that tumors resistant to RAF/MEK inhibition may gain immune-evasive characteristics. Another study stratified tumor samples on baseline PD-L1 levels and found that the PD-L1^+^ subgroup had decreased PD-L1 expression following RAF/MEK inhibitor treatment, possibly due to the overall absence of tumor-infiltrating lymphocytes in progressed samples or a switch to other resistance pathways, such as immunosuppressive cytokine release [51]. In contrast, the baseline PD-L1^−^ subgroup showed a significant rise in PD-L1 expression from RAF/MEK inhibitor pre-treatment to progression despite the increase in the tumor-infiltrating lymphocytes, suggesting T-cell exhaustion is a potential mechanism for immune evasion under RAF/MEK inhibition [51]. Though the two subgroups showed no significant difference in clinical outcome with BRAF/MEK inhibition, these observations may suggest differential benefits for patients based on initial PD-L1 expression levels if immunotherapy is added to the treatment. Collectively, analyses of progressed samples on RAF/MEK inhibition from both studies imply that acquired resistance to targeted inhibition may impair subsequent treatment response to immunotherapy, indicating that administration of immunotherapy prior to targeted inhibitors (IT→TT) may be more beneficial for patients.

Recently, Phadke and colleagues tested the sequence IT→TT, which they defined as two doses of anti-PD-1 every five days followed by daily dabrafenib/trametinib treatment in a BRAF-mutant murine melanoma model [52]. The IT→TT treatment strategy led to a more effective and durable anti-tumor response than IT or dabrafenib-trametinib alone [52]. Furthermore, treatment with TT until tumor regression followed by IT leads to slower tumor growth than TT→IgG control, but not significant tumor regression as observed in the case of IT→TT [52]. They further discovered that the IT→TT sequence is associated with the most significant increase in immune cell influx compared to monotherapies or TT→IT sequence, including the infiltration of CD4^+^ and CD8^+^ T cells [52]. In addition, tumors receiving the IT→TT sequence showed reduced expression of exhaustion markers (e.g., TIM3) on the tumor-infiltrating CD8^+^ T cells, decreased MDSC accumulation, and elevated melanoma antigen expression [52]. A study by Haas and colleagues provided further evidence in melanoma that immunotherapy should be administered before resistance to RAF/MEK inhibition develops. They found that tumors with acquired resistance to RAF inhibitors with and without MEK inhibition also acquire cross-resistance to immunotherapy mediated by an immunosuppressive tumor microenvironment [53]. Furthermore, tumors exhibit low T cell infiltration even when experimentally providing potent tumor antigens, impaired T-cell function, diminished CD103^+^ DCs, and enhanced signaling pathways associated with immune evasion. Notably, induction of CD103^+^ DC responses reversed the immunosuppressive phenotype of TT-mediated cross-resistance and improved response to subsequent IT treatment [53]. In spite of these data, for BRAF mutant melanoma patients with large bulky tumors, BRAF/MEK inhibitors are frequently used to reduce tumor size before initiating immune checkpoint inhibitor. 

The RAS inhibitor rigosertib has been shown to block AKT activation and synergize with anti-PD-1/anti-CTLA-4 in B16F10 (NRAS^WT^BRAF^WT^) and YUMM3.3 (BRAF-mutant) melanoma preclinical models [54]. Remarkably, ICI priming followed by subsequent rigosertib and ICI combination therapy improved responses in B16F10 (NRAS^WT^BRAF^WT^) tumors previously resistant to ICI + dabrafenib/trametinib (BRAF/MEK inhibition) treatment. The rigosertib-ICI combination was also well-tolerated in mice [54]. Mechanistically, rigosertib upregulated CD40 expression on melanoma cells, promoted immunogenic cell death, and increased DC enrichment and T-cell responses in the tumor microenvironment. These events, in turn, sensitized tumors to ICI treatments [54]. In addition, the CD40 expression level has been shown to correlate with markers of antigen presentation and type-I T-cell responses, which are associated with a better OS in a pan-cancer study and therapeutic efficacy of ICI in several melanoma trials [55]. However, it is currently unclear which cell population contributes to increased CD40 expression within the tumor. 

Multiple clinical trials are ongoing to evaluate first-line immunotherapy and targeted therapy in patients with BRAF^V600^-mutant metastatic melanoma. In the phase III DREAMSeq trial (NCT02224781), patients first received nivolumab/ipilimumab (IT) or dabrafenib/trametinib (TT), and then switched to the alternative treatment group upon disease progression. Treatment response was superior in the first-line IT cohorts, with a 2-yr overall survival (OS) rate of 72% vs. 52% and a longer duration of response (DOR) [56]. The SECOMBIT trial (NCT02631447) divided patients into three groups: A) TT (encorafenib/binimetinib) until progression followed by IT (ipilimumab/nivolumab), B) IT until progression followed by TT, and C) a sandwich schedule in which patient received TT for eight weeks, then IT until progression, followed by TT until progression [1]. Similar to the studies mentioned above, IT→TT (Arm B) and the sandwich schedule (Arm C) exhibit superior OS and PFS at years 1, 2, and 3 when compared with TT→IT [1].

However, one also must be cautious about possible severe side effects of sequential treatment with ICIs and targeted kinase inhibition. For example, Dimitriou and colleagues reported that two patients with BRAF^V600^-mutant melanoma developed cytokine release syndrome (CRS) after switching to BRAF/MEK inhibitors upon progression on anti-PD-1 ± anti-LAG-3 antibodies [57]. Thus, despite the promising efficacy of IT→TT, careful evaluation and optimization of the safety profile and patient selection strategies based on biomarker identification for responsiveness are still urgently in need.

In summary, sequential treatment with immunotherapy followed by targeted therapy, or the innovative sandwich schedule which administers immunotherapy before resistance to targeted therapy develops, may offer a more efficacious solution to maximize the synergistic benefit between targeted inhibition and immunotherapy. More survival and safety data and biomarker analysis from the ongoing clinical studies, such as SECOMBIT and DREAMSeq, are needed for future evaluation and trial design.

## 2. Quadruplet Combination of PI3K, RAF/MEK Inhibition, and Immunotherapy

Given the combinatory potential of PI3K/AKT/mTOR and MAPK/MEK/ERK inhibition and synergy with immunotherapy, quadruplet combination therapy may represent a future therapeutic approach, especially for melanoma and TNBC patients harboring mutations or endogenous activation in both MAPK and PI3K pathways (Figure 1). Although clinical research with such combinations is rare, preclinical studies provide interesting insights. In mouse melanoma models with BRAF^V600E^ mutation and PTEN loss, Deken and colleagues showed that PI3Ki + BRAFi + MEKi (triplet therapy) resulted in slightly lower T cell infiltration into tumors when compared with BRAFi + MEKi (doublet therapy) alone, though both groups had significantly increased infiltration compared to untreated mice [58]. While triplet therapy + anti-PD1 showed a significant reduction in tumor size compared with triplet therapy + IgG control, it was slightly less optimal than BRAFi + MEKi + anti-PD1 [58]. Thus, while concurrent quadruplet combination does not seem to bring synergistic effects over MAPK inhibition + ICI combination, it does not impede the benefit, leading the authors to propose that the additional PI3K/AKT/mTOR pathway targeting may be an option for patients with tumors resistant to MAPK inhibition [58]. Well-designed sequential administration of the treatment strategies, such as first-line immunotherapy followed by targeted inhibitors, or inhibitors targeting other immunosuppressive pathways (CXCR2, IDO, etc.) to overcome resistance [59], might prolong the durability of the response and minimize toxicity. 

### 2.1. Novel Pharmacological Inhibitors of PI3K and MAPK Pathways 

Several novel pharmacological inhibitors of PI3K and MAPK pathways are under development. As these new targeted inhibitors enter clinical trials, they could provide a wider toolbox to design combination treatments with enhanced efficacy, tolerability, and specificity. For instance, GDC-0077 preferentially induces HER2-mediated degradation of mutant PI3Kα, resulting in sustained inhibition of phospho-AKT, despite the release of negative feedback of upregulation in receptor tyrosine kinase signaling [60]. In addition, GDC-0077, compared with other PI3K inhibitors, shows stronger potency against PIK3CA-mutant cell lines in vitro and leads to greater tumor regression in PIK3CA-mutant xenograft models [60]. Thus, GDC-0077 may offer exciting opportunities to explore more tolerable and efficacious combinations in melanoma and breast cancer patients harboring PIK3CA mutation [60]. A compelling alternative to small molecule inhibitors, proteolysis-targeting chimeras, manipulate the cellular ubiquitin-proteasome system for targeted polyubiquitination and degradation of protein of interest. MS21, a novel AKT degrader, shows efficient degradation of phospho-AKT and a reduction in downstream signaling [61]. Compared with ATP competitive inhibitor capivasertib, MS21 leads to superior growth inhibition and maintains durable suppression of the AKT pathway [61]. Notably, MS21 also exhibits enhanced anti-proliferative effects in cell lines with mutations in the PI3K-PTEN pathway [61]. While both KRAS and BRAF mutations are associated with resistance to MS21, a combination of MEK inhibition and MS21 effectively overcame resistance in cells with both PI3K and RAS mutations [61]. Another potential pathway of interest to combat intrinsic or acquired resistance to immunotherapy and BRAF/MEK1/2 inhibitors is the MEK/ERK5 pathway (Figure 2). Genetic and pharmacological inhibition of ERK5 in melanoma cells leads to irreversible cellular senescence mediated by p21 and suppresses resistance to BRAF and MEK1/2 inhibition in vitro [62,63]. In TNBC, targeting ERK5 inhibits cell cycle progression, promotes apoptosis, and enhances tumor cell sensitivity to chemotherapy [64]. However, potent and selective inhibitors of MEK5/ERK5 are still under development and need continued optimization.

### 2.2. Biomarkers and Genetic Characteristics for Patient Selection and Stratification

With the complexity of the mechanisms and synergistic interactions of multiple therapeutic agents, biomarker and mutational analysis are essential to the selection of patients who would optimally respond to the treatments. Currently, predictive biomarkers to assess sensitivity to immunotherapy include tumor mutational burden (TMB), lactate dehydrogenase (LDH) level, and immunologic factors such as PD-L1 expression, interferon-γ (IFN-γ) gene signature, CD8^+^ cell infiltration in tumors, and the neutrophil-to-lymphocyte ratio (NLR) [65]. Biomarker analysis from COMBi-I (triplet combination of spartalizumab, dabrafenib, and trametinib) shows that low TMB and high IFNγ gene expression are prognostic for prolonged relapse-free survival and may help identify responsive patients [66]. The IMspire150 trial (triplet combination of atezolizumab, vemurafenib, and cobimetinib vs. doublet targeted inhibitors for BRAF^V600^ mutant melanoma) identified LDH level as the primary determinant of PFS in the atezolizumab arm [65]. In patients with normal LDH, the PFS benefit of atezolizumab is most associated with a strong IFNγ gene signature and the PD-L1^+^ subgroup [65]. In patients with elevated LDH, PFS benefit for atezolizumab is most associated with high TMB and the PD-L1^-^ subgroup, who are often excluded from single ICI treatment [65]. High CD8^+^ infiltration is also a favorable marker for PFS and DOR benefits in the atezolizumab triplet group [65]. The patient subgroup with the best outcomes from triplet combination is normal LDH and high IFNγ gene signature (2-year PFS = 59%), while patients with elevated LDH and low TMB had the poorest outcomes (2-year PFS < 10%) regardless of treatment [65]. The results from IMspire150 demonstrate that biomarkers identified in the single-agent immunotherapy setting may also be predictive for combination treatment. At the same time, it signifies the necessity to look at composite biomarkers and layered stratification.

Preclinical studies and clinical trials often use PIK3CA/AKT1/PTEN status as stratifying markers to target patients for PI3K/AKT/mTOR inhibition [67]. While genetic and protein alteration of PIK3CA/AKT1/PTEN constitutes an important mechanism for PI3K pathway aberrations, the recent biomarker analysis from the FAIRLANE trial (paclitaxel + ipatasertib/placebo before surgery in early TNBC patients) showed that the phospho-AKT level might present a more direct predictive value for the response to PI3K inhibition [68]. High baseline phospho-AKT level is significantly correlated with the higher clinical benefit of ipatasertib, even in patients without PI3KCA/AKT1/PTEN alterations [68]. Increased levels of phosphorylated MEK1/2 and ERK1/2 are also observed in phospho-AKT high samples, indicating the hyperactivation of the MAPK/MEK/ERK pathway and the potential benefit of co-targeting the RAF/MEK pathway in these patients [68].

## 3. Conclusions

In conclusion, the combinatorial and sequential treatment of RAS/PI3K/RAF-targeted therapies after first-line immunotherapy may extend the anti-tumor response for TNBC and melanoma patients, especially those who harbor alterations in both the PI3K/AKT/mTOR and MAPK/MEK/ERK pathways. The recent development of novel pharmacological inhibitors in the PI3K/AKT/mTOR and MAPK/MEK/ERK pathways and biomarker research could pave the ways for more well-designed studies that investigate the tolerability and efficacy of this treatment scheme.

## Figures and Tables

**Table 1:** Ongoing clinical trials combining immune checkpoint inhibitors with inhibitors of the PI3K/AKT/mTOR pathway or MAPK pathway in melanoma and TNBC.

Identifier	Phase	Combination	Drug Names	Indications
NCT01902173	I/II	AKTi + RAFi + MEK1/2i	Uprosertib Dabrafenib Trametinib	Stage IIIC-IV BRAF mutant Cancer
NCT04177108	III	AKTi + α-PDL1 + Chemo	Ipatasertib Atezolizumab Paclitaxel	Locally advanced unresectable or metastatic TNBC
NCT03395899	II	AKTi + α-PDL1, orMEK1/2i + α-PDL1	Ipatasertib Atezolizumb Cobimetinib	Untreated operable ER+ HER2- breast cancer
NCT03424005	Ib/II	AKTi + α-PDL1	Ipatasertib Atezolizumab	Locally advanced unresectable or metastatic TNBC
NCT03742102	Ib/II	AKTi + α-PDL1 + Chemo	Capivasertib Durvalumab Paclitaxel	Metastatic TNBC
NCT02858921	II	B-RAFi + MEK1/2i +α-PD1	Dabrafenib Trametinib Pembrolizumab	BRAF mutant resectable stage II melanoma
NCT04835805	Ib	pan-RAFi + MEK1/2i +α-PDL1	Belvarafenib Cobimetinib Atezolizumab	NRAS-mutant advanced melanoma
NCT03625141	II	MEK1/2i + α-PDL1 +BRAFi	Cobimetinib Atezolizumab Vemurafenib	BRAF^V600^ wild-type or mutant melanoma with central nervous system metastases
NCT04722575	II	Neoadjuvant BRAFi +MEK1/2i + combinationor adjuvant α-PDL1	Vermurafenib Cobimetinib Atezolizumab (neoadjuvant vs. adjuvant)	High-risk, surgically resectable BRAF-mutated melanoma
NCT03554083	I	BRAFi + MEK1/2i +αPD-L1	Vemurafenib Cobimetinib Atezolizumab (neoadjuvant + adjuvant)	High-risk, stage III melanoma
NCT02910700	II	BRAFi + MEKi + αPD1	Dabrafenib Nivolumab Trametinib Binimetinib Encorafenib	Metastatic melanoma

TNBC, triple-negative breast cancer. AKTi, AKT inhibitor. MEKi, MEK inhibitor. RAFi, RAF inhibitor.

**Table 2:** Results from clinical trials combining immune checkpoint inhibitors with inhibitors of the PI3K/AKT/mTOR pathway or MAPK pathway in melanoma and TNBC.

Identifier	Phase	Combination	Drug Names	Indications	Results
NCT03742102 (BEGONIA)	Ib/II	AKTi + αPDL1 + Chemo	Capivasertib Durvalumab Paclitaxel	Metastatic PD-L1+ TNBC	ORR = 16/30 (53.3%) G3/4 trAE =22/30 (73%)
NCT03961698 (Mario-3)	II	PI3Kγi + α-PDL1 + Chemo	Eganelisib Atezolizumab Nab-paclitaxel	Locally advanced unresectable or metastatic TNBC	ORR = 21/38 (55.3%)
NCT02908672 (IMspire150)	III	B-RAFi + MEK1/2i → B-RAFi + MEK1/2i + α-PDL1	Vemurafenib Cobimetinib Atezolizumab	Advanced unresectable BRAF^V600E^ melanoma	PFS = 15.1mo vs. 10.6mo G3/4 trAE = 79% vs. 73% n = 514
NCT02130466 (KEYNOTE-22)	I/II	B-RAFi +MEK1/2i + α-PD1	Dabrafenib Trametinib Pembrolizumab	Unresectable or metastatic BRAF^V600E^ melanoma	PFS = 16.9mo vs. 10.7mo G3-5 trAE = 58% vs. 25% n =120
NCT02967692 (COMBI-i)	III	B-RAFi + MEK1/2i + α-PD1	Dabrafenib Trametinib Spartalizumab	Unresectable or metastatic BRAF^V600E^ melanoma	PFS = 16.2mo vs. 12.0mo G3-5 trAE = 55% vs. 33% n = 532
NCT02224781 (DREAMSeq)	III	α-PD1 + α-CTLA4 (IT) or BRAFi + MEK1/2i (TT) first, switch treatment upon progression	Nivolumab-Ipilimumab Dabrafenib-trametinib	Metastatic BRAF^V600E^ melanoma	2-yr OS = 72% vs. 52% n = 265
NCT02631447 (SECOMBIT)	II	B-RAFi MEK1/2i (TT) or α-PDL1 + α-CTLA4 (IT) first, switch treatment upon progression, or TT(8wks) + IT until progression + TT	Nivolumab-Ipilimumab Encorafenib-Binimetinib	Metastatic BRAF^V600E^ melanoma	2-yr OS = 62% vs. 73% vs. 69% 3-yr OS = 53% vs. 63% vs. 60% G3/4 trAE = 28% vs. 54% vs. 32% n = 251

ORR, objective response rate. trAE, treatment-related adverse events. OS, overall survival. PFS, progression-free survival.

## Data Availability

Publicly available datasets were analyzed in this study. This data can be found here: cBioportal.org (accessed on 2 April 2022).
